# Detection of atmospheric radon concentration anomalies and their potential for earthquake prediction using Random Forest analysis

**DOI:** 10.1038/s41598-024-61887-6

**Published:** 2024-05-31

**Authors:** Mayu Tsuchiya, Hiroyuki Nagahama, Jun Muto, Mitsuhiro Hirano, Yumi Yasuoka

**Affiliations:** 1 Department of Earth Science, Graduate School of Science, Tohoku University, 6-3 Aramaki-Aza-Aoba, Aoba-ku Sendai, 980-8578 Japan; 2 Radioisotope Research Center, Kobe Pharmaceutical University, 4-19-1 Motoyamakitamachi, Higashinada-ku, Kobe, 658-8558 Japan; 3 School of Engineering, Utsunomiya University, 7-1-2 Yoto, Utsunomiya, 321-8585 Japan

**Keywords:** Natural hazards, Solid Earth sciences

## Abstract

Various anomalies occurring before earthquakes are currently being studied to predict seismic events, with one of them being the radioactive element radon (^222^Rn). Radon concentrations in the soil, water, and atmosphere fluctuate in response to crustal movement. Recent research has statistically detected anomalies by analyzing the fluctuations in radon concentrations before earthquakes and conducting quantitative evaluations of radon. However, the method used to determine the parameters in the analysis was problematic. Therefore, in this study, we compared observed atmospheric radon concentration data with predicted values based on typical annual patterns using Random Forest analysis. We conducted a more objective analysis by employing this method and statistically determining anomalies using thresholds. This analysis was conducted using atmospheric radon concentration observation data obtained at Kobe Pharmaceutical University (KPU) before the 1995 Kobe Earthquake, and ionization currents emitted when radon decays were obtained at Fukushima Medical University (FMU) before the 2011 Tohoku-oki Earthquake. Consequently, before the major earthquakes occurred at both locations, the difference between the predicted and observed values exceeded the standard deviation by a factor of three. These results indicate the potential of Random Forest analysis to identify anomalies in atmospheric radon concentrations before earthquakes occur.

## Introduction

Currently, various researchers are searching for the precursor phenomena of earthquakes, with a particular focus on the study of the radioactive element radon^1^. Radon (^222^Rn) is an element with an atomic number of 86 and a half-life of 3.82 days. It is a noble gas and exhibits behavior similar to that of an ideal gas. Previous studies have revealed that radon concentrations in the soil, water, and atmosphere vary in response to crustal movement. In the Gulf of Corinth in Greece, atmospheric radon concentration suddenly decreased immediately before an earthquake^2^. Concerning underwater radon concentrations, the radon in groundwater decreased three months before the Izu-Oshima-kinkai Earthquake, which occurred on January 14, 1978, and experienced a sharp decline five days before the earthquake^3^. Moreover, the radon concentration in groundwater increased several months before the 1995 Kobe Earthquake, peaking nine days before the earthquake^4^. Atmospheric radon concentrations have been shown to vary in response to crustal strain in the range of ${10}^{-6}$ to ${10}^{-8}$^5^. Thus, among water, soil, and air, atmospheric radon concentration serves as an indicator of fluctuations in response to even small strains.

In the aforementioned atmospheric radon concentration studies, the data exhibited seasonal cycles^6^. These seasonal fluctuations can be represented by sinusoidal waves^7,8^. Previous research has aimed to detect anomalies in atmospheric radon concentrations by identifying and removing seasonality from observational data during earthquake-free periods and then analyzing the residuals to correlate to earthquakes^7,9^. The average radon concentrations and the smoothed atmospheric residuals showed anomalies prior to the Kobe Earthquake of 1995^10^. Fluctuations in atmospheric radon concentration in response to tidal loading have also been observed during this period^11^. A similar anomalous increase was also observed before and after the 2011 earthquake in northern Wakayama Prefecture^9^. In contrast, before the 2018 Northern Osaka Earthquake, an abnormal decrease in atmospheric radon concentration associated with quiescent seismic activity was observed^12^. Thus, abnormal phenomena in atmospheric radon concentrations before earthquakes are not limited to increases but also include abnormal decreases. However, in these analyses of atmospheric radon concentrations, the magnitudes of the residuals in atmospheric radon concentrations were influenced by the determination of the sine curve representing seasonal variations, leading to uncertainties in anomaly detection.

To address this issue, Iwata et al. (2018) conducted anomaly detection using singular spectrum transformation (SST) without removing seasonal variations by directly utilizing observational data^13^. SST analysis can detect anomalies in periodic data, eliminating the need for preset sine curves that reflect seasonal variations. This advantage allows anomaly detection even in data where the underlying physical processes are not explicitly known. Using this approach, Iwata et al. (2018) analyzed atmospheric radon concentrations observed before the 2011 Tohoku-oki Earthquake. By comparing changes in the cumulative seismic moment with the rate of change in atmospheric radon concentrations, which was determined using SST, they were able to quantitatively evaluate anomalies. The results of this study reveal a statistically significant correlation between the detected anomalies in atmospheric radon concentrations and changes in the cumulative seismic moment. Furthermore, an increase in the rate of change was observed before the 2011 Tohoku-oki Earthquake. This period coincides with various anomalies, such as slow-slip events in the Japan Trench^14^. Additionally, a decrease in groundwater level and temperature in the wells was observed near the northern end of the main rupture region of the earthquake^15^. In addition, when seismic activity was analyzed using natural time analysis, the entropy changes in seismic motion reached a minimum of approximately three months before the Tohoku-oki Earthquake^16^. The alignment of these anomalies with crustal movements and other anomalies not limited to radon made the occurrence of preseismic anomalies more evident. However, SST analysis still has uncertainties in anomaly detection, owing to the influence of the parameters used in the analysis and the ambiguity in determining the criteria for considering an increase in the rate of change as abnormal.

**Figure 1:**
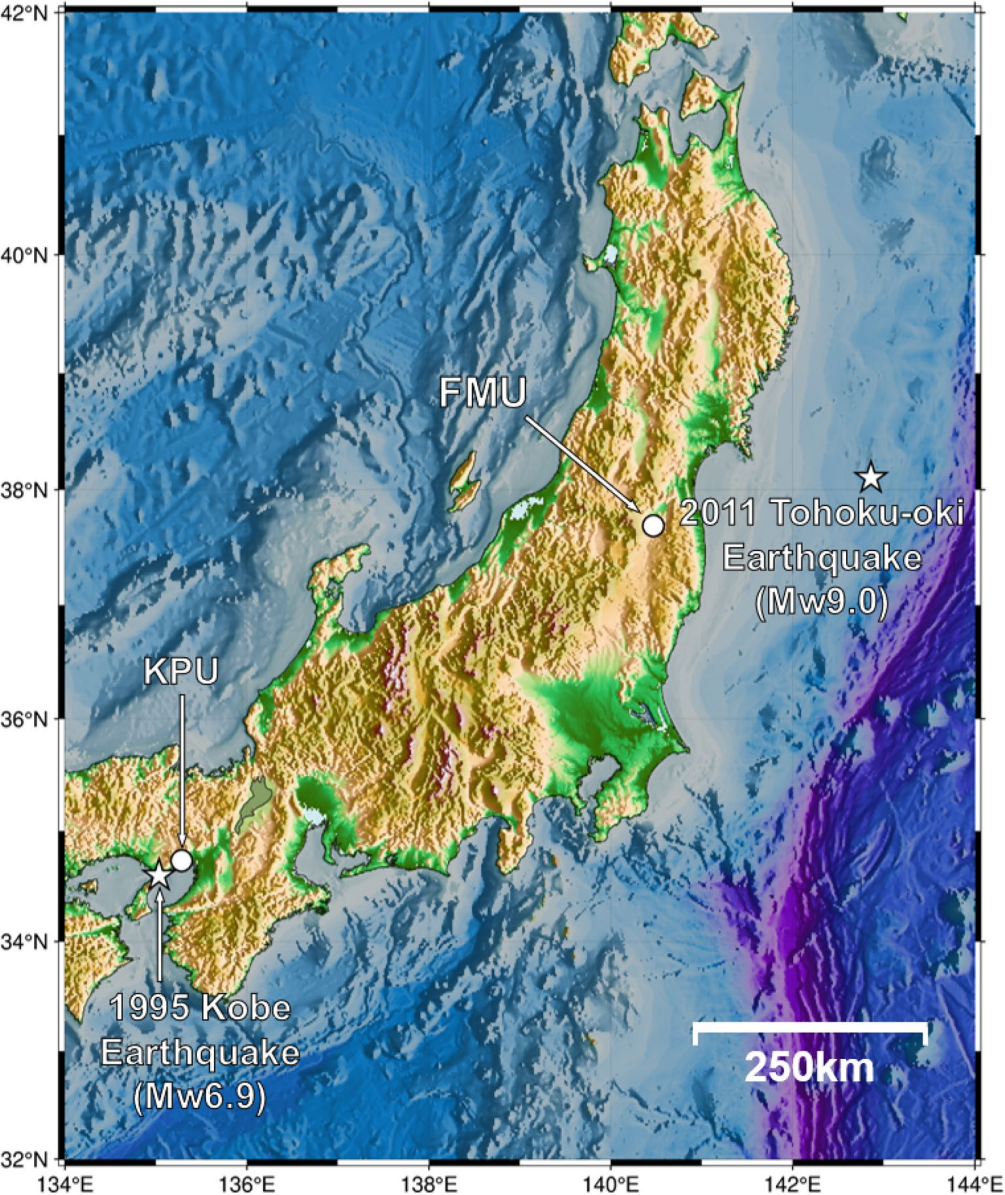
Map showing the locations where ionization current values were measured and the epicenters of the great earthquakes. The circles and stars indicate the locations of the observational site (FMU and KPU) and the epicenters (the 2011 Tohoku-oki Earthquake and the 1995 Kobe Earthquake), respectively. This map was created using PyGMT^40^.

To address the SST problem, attempts were made to detect anomalies in atmospheric radon concentrations before the Kobe and Tohoku-oki Earthquakes (Fig. 1) using Random Forest analysis, as such anomalies have been reported in previous studies. These two earthquakes were used in this study because observational data were available for a long period before the mainshock, and previous studies have made detailed comparisons with seismic events. Random Forest analysis is a type of machine learning that utilizes models created from learned data to predict or classify out-of-sample data without an arbitrary selection of parameters (see Methods section for details). Furthermore, adopting a 3σ threshold as the criterion for considering anomalies resolves the ambiguity in anomaly determination^17^. This analysis targeted the detection of anomalies with greater objectivity than conventional anomaly detection.

## Results and discussion

**Figure 2:**
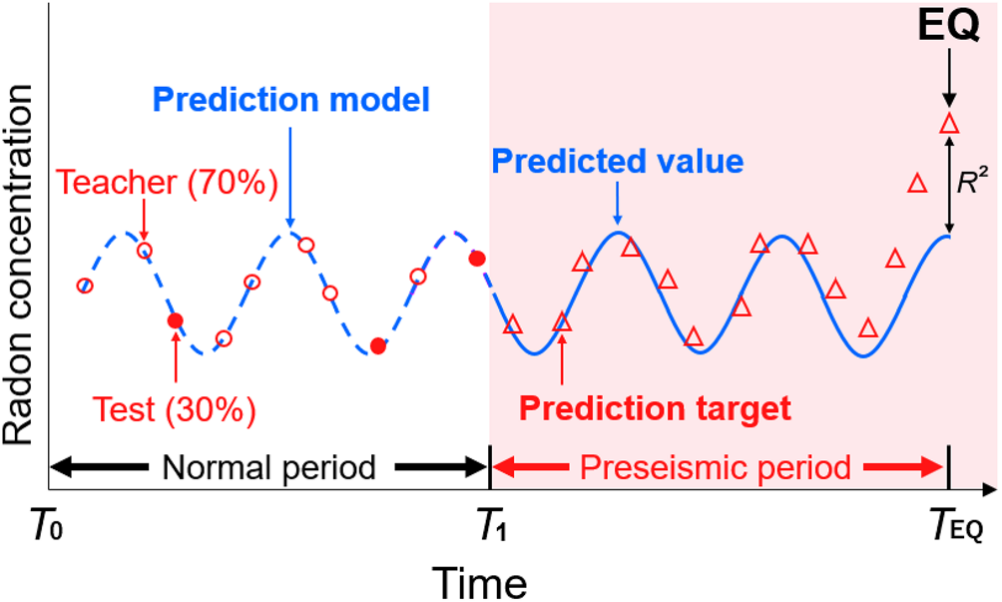
The schematic image of the Random Forest prediction model constructed in this study. The normal period is the period of time during which no major earthquakes have occurred in the region/period of interest. The red dots represent test data points, comprising 30% of the observed atmospheric radon concentrations or ionization current during normal periods, while the white-outlined red dots represent teacher data points, comprising 70% of the observed atmospheric radon concentrations during normal periods. The red triangles denote observed atmospheric radon concentrations or ionization current during the preseismic period. The blue dashed line represents the prediction model created by training on the observation data (white-outlined red dots), while the blue solid line shows the predicted values for the preseismic period based on that model. The accuracy of the model was calculated using the *R*^2^ of the coefficient of determination moored from the predictions and observations. EQ: earthquake, ${T}_{EQ}$: earthquake day.

To create a Random Forest prediction model, the observation period without particularly large earthquakes was designated as the normal period, and the subsequent periods of earthquakes were considered the preseismic period (Fig. 2). The data were split into normal and preseismic periods for Kobe Pharmaceutical University (KPU) and Fukushima Medical University (FMU) (Table 1). The KPU had a deficit of approximately one month in the observation data after the earthquake due to the damage caused by the Kobe Earthquake. The ionization current at the FMU increased after the Tohoku-oki Earthquake due to the high environmental radiation caused by the accident at the Fukushima Daiichi Nuclear Power Plant^18^. Therefore, observational data up to immediately before the earthquake was used in this study. Based on the data from the normal period, we created prediction models and then validated the accuracy of the generated predictive model by calculating the coefficient of determination. The date of observation was used as an explanatory variable, and the observed data were used as objective variables. Furthermore, to assess the feasibility of performing anomaly detection in real time and explore whether it can be analyzed using simpler methods, we utilized the observed data for training without excluding outliers.

**Table 1:** Locations of observational sites and periods of the ionization current data in the analysis.

Observational site	Location	Normal period (Teacher data)	Preseismic period
Kobe Pharmaceutical University (KPU)	N34 ˚43 ′59" E135˚17′0"	January 1, 1984–December 31, 1988	January 1, 1990–January 16, 1995
Fukushima Medical University (FMU)	N37 ˚41 ′25" E140˚28′15"	February 13, 2002–December 31, 2007	January 1, 2008–March 10, 2011

### The case of KPU

**Figure 3:**
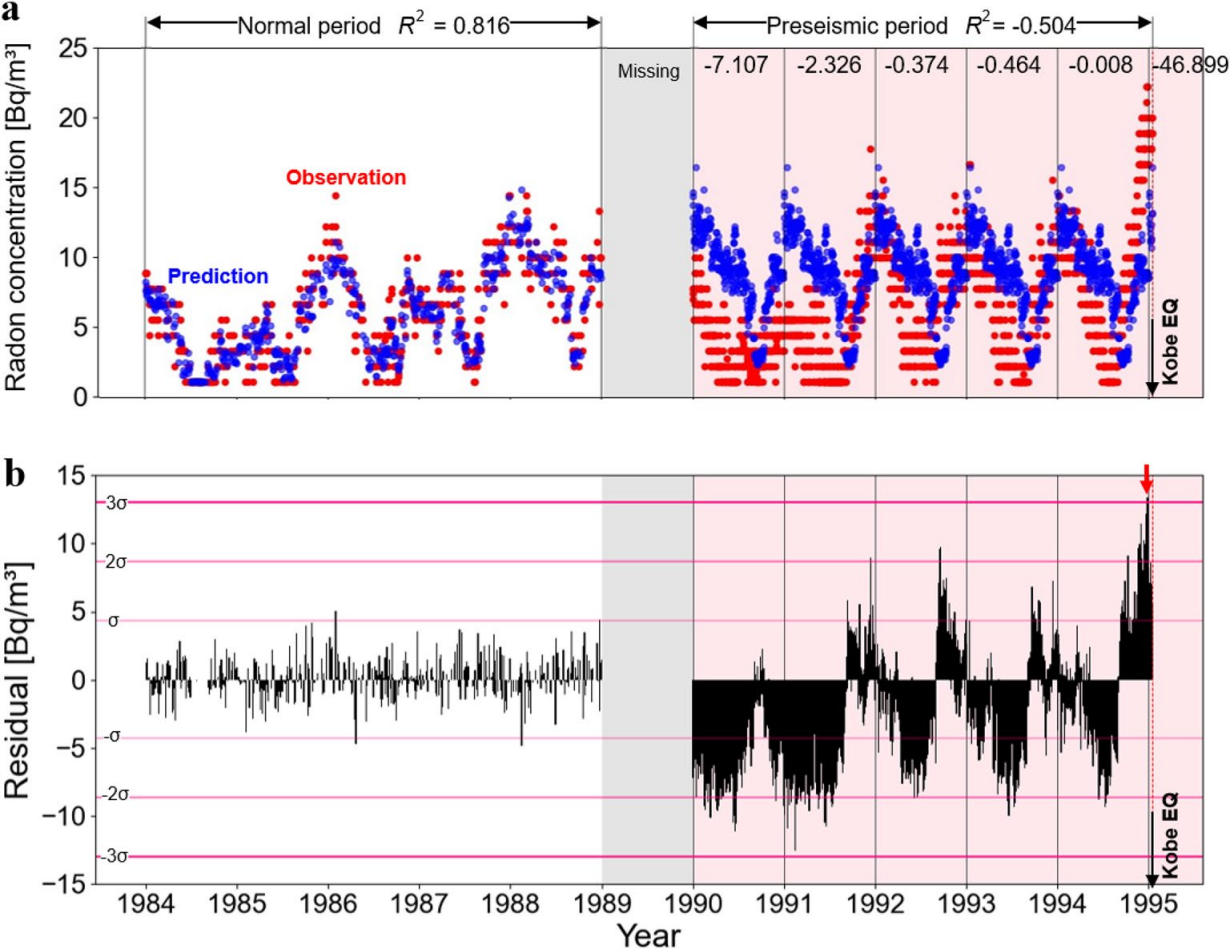
(**a**) Prediction model and results in KPU. Red dots indicate actual ionization current values and blue dots indicate predictions obtained by Random Forest analysis. The coefficients of determination for each period are shown at the top. (**b**) Discrepancy between observed and predicted ionization current values at KPU. Redish horizontal lines represents ± σ, 2σ and 3σ calculated from the standard deviation of the difference. The red arrow points to December 23rd and 28th, 1994, when the difference significantly exceeded 3σ^17^.

To analyze the KPU, atmospheric radon concentration data from 1984 to 1995 was utilized, and a prediction model was developed using data from 1984 to 1988. This model is illustrated in Fig. 3a. As shown in Fig. 3a, the shapes of the graphs for the predicted and observed values were generally consistent. The coefficient of determination for this period was 0.816, indicating high reproducibility of the prediction model. Using this prediction model, we were able to replicate changes in the atmospheric radon concentration that occurred during the preseismic period. Figure 3a shows that during the preseismic period, there were many instances in which the observed values fell below the predicted values. In particular, a trend towards lower values was evident from 1990 to 1991. From 1992 to 1993, there were a few instances in which the observed values deviated significantly from the predicted values. However, from mid-1994 to early 1995, the predicted values significantly exceeded the observed values. To quantitatively evaluate fluctuations during the preseismic period, we calculated the coefficient of determination *R*^2^ for the period (1990–1995). The overall *R*^2^ value for the preseismic period was − 0.504. Until 1991, the values remained consistently low, with 1990 at − 7.107 and 1991 at − 2.326, both falling below − 1. Leading up to 1994, there was a gradual increase in values, with 1992 at − 0.374, 1993 at − 0.464, and 1994 at − 0.008. However, in 1995, the value dropped significantly to − 46.899.

As shown in Fig. 3b, an examination of the residual values between the observed and predicted values revealed variations in the atmospheric radon concentration during the normal and preseismic periods. As shown in Fig. 3a, the period from 1990 to 1991 exhibited values significantly below the predicted values. From 1992 to 1994, there were numerous instances in which the observed values fell below the predicted values during spring and summer and exceeded the predicted values during autumn and winter. From mid-1994 on, there was an extended period during which the observed values consistently exceeded the predicted values.

As mentioned previously, there was a period of low atmospheric radon concentrations from 1990 to 1991. There have been instances of seismic quiescence before significant earthquakes; indeed, seismic quiescence was reported before the 1995 Kobe Earthquake^19^. Prior to the 2018 Northern Osaka Earthquake, a decrease in atmospheric radon concentration linked to seismic quiescence was documented. This suggests that, similar to the Northern Osaka earthquake, seismic quiescence occurred in the vicinity of KPU from 1990 to 1991, as reflected by the low atmospheric radon concentration.

From the latter half of 1994 until just before the Kobe Earthquake, there was a period when predicted values were much higher than observed values. Between 1992 and 1994, the difference between the observed and predicted values was small, and the graph indicates that seasonal variations in atmospheric radon concentration were evident in the observed values. Consequently, it is believed that from the end of 1994, there was a noticeable divergence in trends between the normal and preseismic periods. From Fig. 3b, the difference between the observed and predicted values was calculated, and its standard deviation was set as σ. 2σ showed no correspondence with the earthquake and no continuous variation. However, the difference exceeded 3σ from December 23 to 28, 1994, when the earthquake was imminent^17^.

The increase in radon concentration prior to the Kobe Earthquake can be attributed to changes in the stress field of the area. In the vicinity of KPU, a transition from a contractive to an extensional strain field has been reported since mid-1994^20^. It has been revealed that an extensional stress field promotes the release of carbon dioxide from near-surface fluids^21^. Therefore, it is likely that the transition from the compressional stress field to the extensional stress field enhances radon emanation, leading to a significant increase in atmospheric radon concentration exceeding 3σ at the end of 1994. Thus, the significant increase in atmospheric radon concentrations detected at the end of 1994 in the Random Forest analysis in this study is considered an anomaly that has been confirmed in previous studies. This confirms the robustness of the prediction and anomaly-detection methods based on Random Forest analysis used in this study.

### The case of FMU

**Figure 4:**
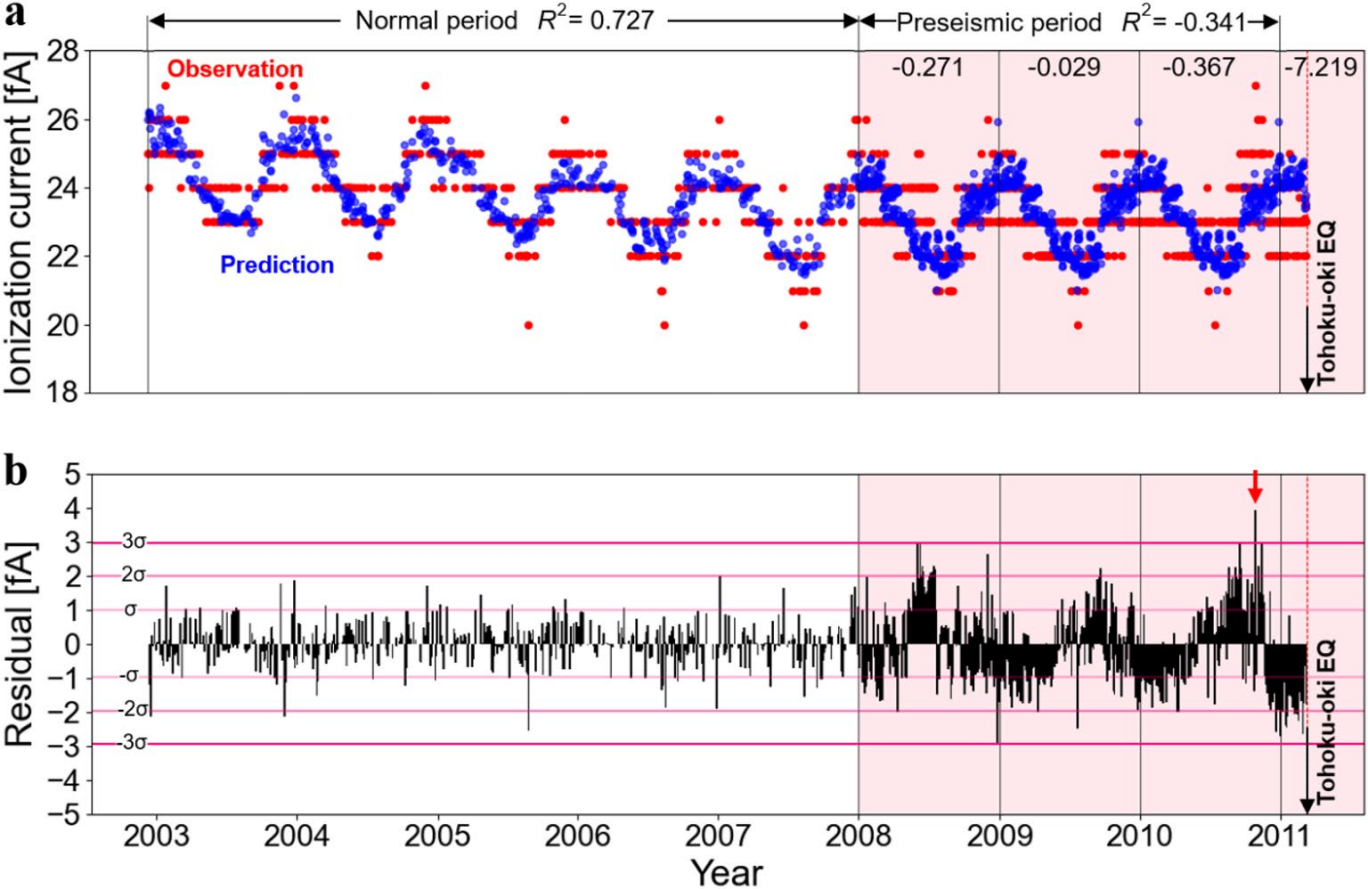
(**a**) Prediction model and results in FMU. Red dots indicate actual ionization current values and blue dots indicate predictions obtained by Random Forest analysis. The coefficients of determination for each period are shown at the top. (**b**) Discrepancy between observed and predicted ionization current values at FMU. Redish horizontal lines represents ± σ, 2σ and 3σ calculated from the standard deviation of the difference. The red arrow points to October 28, 2010, when the difference significantly exceeded 3σ^17^.

During this application to the FMU, we utilized data consisting of the ionization current measured at the RI facility of the FMU. The ionization current values can be linearly converted to atmospheric radon concentrations (see Eq. (1) in the “Methods” section for details). The forecasting was done using data from 2008 to 2011, while the prediction model was developed using FMU data from 2002 to 2007. Figure 4 displays the model and forecast results after 2008. As shown in Fig. 4a, the observed values exhibited periodicity resembling a sinusoidal curve with peaks in both the normal and preseismic periods. Additionally, it can be noted that from 2008 to mid-2010 the shape of the predicted values generally matched the shape of the observed values. However, in the latter half of 2010, a shift in the peak seasonal variation became apparent. Given that the observed values demonstrate a periodicity similar to a sinusoidal curve throughout the normal and preseismic periods, this characteristic is consistent with previous research^7^. The presence of minima and maxima in the vicinity of the peaks indicates that a relatively accurate prediction model was created for the FMU. The coefficient of determination *R*^2^ of this model for the normal period (2003–2008) was 0.727. A high determination value indicates that the prediction model is highly accurate and demonstrates its robustness.

Next, we compared the observed and predicted preseismic ionization current data from 2008 to 2011 using a Random Forest analysis. No phase shift was observed in the seasonal variation of the ionization current between the predicted and observed data from 2008 to mid-2010. However, a phase shift was observed after September 2010. In addition, the predicted values reproducing the normal period show a clear delay in the positive peak values prior to the Tohoku-oki Earthquake. Furthermore, higher ionization currents were observed at the end of 2010, immediately before the earthquake.

The observed variation in the preseismic ionization current data was also recognized in the coefficient of determination *R*^2^ between the prediction model and the observed values. To quantitatively evaluate this variation, we calculated the *R*^2^ before the earthquake (2008–2011) to be -0.341. This indicates that the prediction accuracy during this period was lower than during the normal period (2003–2008). To identify the periods during which the observed values of the ionization current deviated significantly from the predictions based on seasonal variations, the standard deviation of the difference between the predicted and observed values was calculated. *R*^2^ calculated for each year within the preseismic period showed an anomalous variation toward the Tohoku-oki Earthquake. The *R*^2^ values remained somewhat low at − 0.271 in 2008, − 0.029 in 2009, and − 0.367 in 2010, but dropped significantly to − 7.219 in 2011. The significant decrease in the *R*^2^ value in 2011 indicates that the atmospheric radon concentration estimated from the ionization current data deviated significantly from the normal period.

An anomalous variation was also observed in the residual variation of the ionization current (Fig. 4b). Figure 4b shows the difference between the observed and predicted values for the preseismic period in the FMU, with a line drawn at 3σ. From Fig. 4b, it is evident that the residual is generally larger during the preseismic period than during the normal period. In mid-2008, late 2008, and late 2010, the residual notably increased, but particularly by late 2010, it is apparent that the observed ionization current values exceeded 3σ, significantly deviating from the seasonal variation.

From the GNSS time series measured before the Tohoku-oki Earthquake, it was determined that northeastern (NE) Japan underwent east–west deformation from 2003 to 2010^22,23^, with significant eastward deformation occurring in 2008 and 2010^24^. The eastward motion leading to volumetric expansion was caused by an aseismic slip in the Japan Trench^23^. This suggests that similar to KPU, extensional deformation occurred during the preseismic period^21^, leading to an increase in the ionization current during this period. Iwata et al. (2018) conducted cumulative seismic moment calculations simultaneously with SST analysis and reported an increase in the cumulative seismic moment associated with aseismic slip^13^. Radons in soil are released into the atmosphere through soil fissures, and changes in soil fissures due to crustal deformation are expected to sensitively alter the concentration of radon in the atmosphere^25,26^. Based on these observations, Iwata et al. (2018) suggested that the expansion of fractures caused by volumetric expansion in NE Japan due to aseismic slip led to an increase in the rate of radon emanation to the surface^13^. Therefore, the significant increase in atmospheric radon concentration detected in this Random Forest analysis was likely associated with changes in the fracture width due to aseismic slip, which is similar to the findings of Iwata et al. (2018).

## Practical challenges for real-time monitoring of atmospheric radon concentrations

To establish a real-time monitoring network for earthquakes and crustal movement in the future, recent developments in the monitoring networks of atmospheric radon concentrations measured by Radioisotope facilities (RI facilities) across Japan have enabled us to synchronously monitor anomalies related to crustal deformation in Japan using radon concentrations^12^. Muto et al. (2021) pointed out that these measurements reveal that atmospheric radon concentration reflects the average values of radon exhalation and is independent of local heterogeneity in geological and hydrological structures. In the network, the non-parametric analysis used to estimate change points in the time series clearly indicates that the change in atmospheric radon concentration is statistically correlated with the seismic activities in the Kobe Earthquake and Tohoku regions prior to the 2011 Tohoku-oki Earthquake^13^.

Moreover, for real-time monitoring, it is necessary to validate the effectiveness of this method for other earthquakes occurring in various tectonic settings. To validate this method, we applied Random Forest analysis to Wakayama Medical University (WMU) during 2011–2013, where we obtained data on atmospheric radon concentration variations during the earthquake swarm period^9^. The results show that the difference between observed and predicted values before the 2011 and 2013 earthquakes exceeded 3σ (Refer to the supplementary Fig. S1, S2). This indicates that Random Forest analysis of atmospheric radon concentration variations is also effective for earthquake swarms with short cycles. Further analysis is required to demonstrate the versatility of these methods for earthquakes occurring in other regions, including other countries.

Previous studies have shown that seismic activity and weather are influenced by solar activity^27–29^. Because the solar triggering cycle is an 11-year cycle^30^, continuous observation data longer than a multiple of 11 years is required to accurately consider the effects of solar activity in the analysis. To achieve this, further research is essential to verify the validity of the method in various locations, analyze the method using longer-term data, and examine the lead time between anomaly detection and earthquake occurrence. During the periods adopted for the “Teaching Phase” and “Testing Phase,” when local seismicity M6 + is incorporated into the data, the seasonal levels of radon are periodic and seismic events are randomly related to the time scale. Therefore, training, testing, and anomaly detection can potentially be biased by the data used to predict the radon time series. Therefore, it is recommended that radon concentration fluctuations during the seismic quiet period be used in the “Guidance phase” and “Test phase.” With the resolution of these issues, anomaly detection for earthquake prediction has become considerably more important. However, in this study, including M6 + in the catalog did not affect the main results capturing the anomalies of the mainshock, and anomalies were observed before the M7 earthquake. To seek greater accuracy in targeting smaller earthquakes, a future challenge will be to create a new dataset that excludes the confidence of M6 + and reanalyze it. Therefore, the results of the Random Forest analysis of atmospheric radon concentration variations indicate the potential to identify abnormalities in atmospheric radon concentrations before earthquake occurrence, suggesting the possibility of capturing earthquake precursors.

## Conclusion

In this study, we conducted Random Forest analysis at both KPU and FMU to minimize ambiguity for parameter determination using a statistical approach for anomaly detection based on numerical values for atmospheric radon concentration before earthquakes. This allowed for more objective anomaly detection when compared with SST. The results of the Random Forest analysis revealed anomalies in the ionization current data, indicating significant discrepancies between the predicted and observed data during specific periods before the 1995 Kobe Earthquake at KPU and before the 2011 Tohoku-oki Earthquake at FMU.

Consequently, it has become possible to analyze the observed ionization current values as raw data, leading to the potential for real-time anomaly detection in short-term earthquake predictions using atmospheric radon concentrations. The ionization currents were measured at other RI facilities. This study detected anomalies from the observed ionization current data without converting them into atmospheric radon concentrations, suggesting the possibility of promptly detecting anomalies, even without prior knowledge, to convert ionization currents into atmospheric radon concentrations. Additionally, the direct utilization of observational data can simplify the procedures for real-time detection, potentially contributing to the establishment of a nationwide real-time monitoring network for earthquakes and crustal movement using RI facilities. If a prediction model for atmospheric radon concentrations beyond normal periods is established, real-time anomaly detection becomes feasible by comparing the observed atmospheric radon concentration with that of the model, potentially contributing to the development of a crustal deformation-monitoring network.

Future improvements are needed, such as analyzing data from other locations over a longer period of time to validate its effectiveness and excluding the confidence of M6 + from the training dataset to achieve more accurate anomaly detection.

## Method

### Atmospheric radon concentration data

The data used for the analysis were derived from atmospheric radon concentration measurements at KPU before the 1995 Kobe Earthquake (Mw 6.9)^10^ and ionization current data measured at FMU before the 2011 Tohoku-oki Earthquake (Mw 9.0)^13^. Atmospheric radon concentration at KPU was measured with a gas flow-type ionization chamber (Fuji Electric Systems Co., Ltd., Japan; model: NAG513; effective volume: 1.8 × ${10}^{-2}$ m^3^)^5^. Measurements were performed at FMU using another gas flow-type ionization chamber (Aloka Co., Ltd, Japan; model: DGM-101; effective volume: 1.4 × ${10}^{-2}$ m^3^; equipped with ^90^Sr calibration source)^7^. The ionization current data included both the background radiation values and ^90^Sr radiation levels provided by the instrument.

In this study, it was assumed that the ionization current data represented the concentration of radon in the atmosphere through a linear transformation using the following formula^31^:

1 $$\begin{array}{c}C=\left(FIW/eEV\right)\times {10}^{-15}=fI,\end{array}$$

where $C$ is the atmospheric radon concentration, $F$ is the correction coefficient, $I$ is the ionization current, $W$ is the *W*-value of alpha particles in air, $e$ is the elementary charge, $E$ is the alpha energy, $V$ is the effective volume, and $f$ is the conversion coefficient^31^. Records at hourly intervals using the daily minimum values among the data, which were least affected by weather and geographical conditions, were deemed to best represent the daily atmospheric radon concentration^32^. Days with missing data were treated as missing values and data from leap days were excluded.

### Random Forest analysis

Introduced by Breiman^33^ in 2001, Random Forest analysis belongs to the ensemble learning category of machine learning^33^. The main goal is to integrate insights from multiple models to derive definitive answers. In the RF domain of Random Forests, ensemble learning models are created by using a methodology known as bagging. This process generates multiple groups of training data, including duplicates, from the entire training data pool. By creating a large number of decision trees characterized by low accuracy and minimal intermodel correlation, Random Forests effectively prevent overfitting during the machine learning process. Random Forest analysis is versatile and can be used for data classification and regression. In the classification scenario, the final decision was made by majority vote among the results provided by the multiple decision trees. Conversely, in the regression task, the final result is computed by averaging the predictions generated by multiple decision trees. This averaging technique plays an important role in mitigating the effects of the noise inherent in each decision tree. The decision trees generated using the bagging technique adhered to a consistent distribution^34^. This consistency guarantees that the expected value of the mean derived from a collection of X decision trees aligns with the anticipated value of each decision tree. We used Random Forest analysis for multiple regression in this study, which allowed us to forecast data outside of the training data timeframe.

### Creating the prediction model

In this study, we employed multiple regression using a Random Forest in order to forecast future data beyond the training data period. The analyses were performed using Python version 3.8^35^, Scikit-learn^36^, Pandas^37^, and NumPy^38^. To assess the robustness of the Random Forest time-series prediction model, we randomly selected 70% of the data from the normal period for both FMU and KPU to create prediction models. Subsequently, we compared the predicted data with the remaining 30% of the data from the normal period and calculated the coefficient of determination (*R*^2^) to confirm the model’s prediction accuracy (Fig. 2). To construct Random Forest prediction models and perform model fitting, we utilized the observed data as explanatory variables. Previous studies have indicated that atmospheric radon concentrations are temperature dependent^39^ and fluctuate seasonally with the northward and southward movement of the rainy season fronts. Because the teacher data for the Random Forest analysis includes annual variations that reflect seasonal variations due to the movement of such fronts, they do not affect the discrepancy between the observed and predicted values. When creating the model, we executed a Random Forest with an unrestricted depth as well as depths of 5, 10, 15, 20, and 25. The depth with the highest coefficient of determination between observed and predicted values was selected. Consequently, the depth of 10 m had the highest coefficient of determination. Supplementary Table S1 shows the calculated coefficients of determination at each depth.

### Determination coefficient

To assess the agreement between the predicted and observed data, we employed coefficients of determination derived from their differences. The coefficient of determination, denoted as *R*^2^, was calculated based on the predicted value* u*, measured value *v*, and mean value $\overline{v}$ of the measured data *v* as follows^36^:

2 $$\begin{array}{c}{R}^{2}\left(u,v\right)=1-\left\{\sum_{i=0}^{n-1}{\left({v}_{i}-{u}_{i}\right)}^{2}\right\}/\left\{\sum_{i=0}^{n-1}{\left({v}_{i}-\overline{v }\right)}^{2}\right\}.\end{array}$$

If the predicted and observed values were in agreement, then* R*^2^ was equal to 1. If there was a large discrepancy between the predicted and observed values,* R*^2^ had a negative value. If the slopes or waveforms of the actual and predicted data differed, the *R*^2^ value was negative. After evaluating the accuracy of the model by *R*^2^ in this way, the model was applied to predict the ionization current values for the specified data period. The *R*^2^ values were then compared with the model values to explore anomalies in preseismic atmospheric radon concentrations relative to normal conditions. Furthermore, the coefficient of determination of the model was validated through cross-validation by dividing the normal period into five segments. Please refer to Supplementary Table S2 for the respective values.

### Anomaly identification method

In order to detect anomalies in the observed atmospheric radon concentrations and ionization current values, we employed a 3σ criterion. As there is no general definition of anomalies for time-series data of geochemical and geophysical observations, it is necessary to set standards for anomalies suitable for radon observations^17^. We considered that both the amplitude and duration were sufficient to distinguish it from seasonal fluctuations, and defined radon changes that maintained a level of 3σ or higher for a period of one day or more as an anomaly.

First, we subtract the predicted values obtained through Random Forest analysis from the observed values to calculate the residuals. Next, we computed the standard deviation σ of the residuals. Based on the properties of the Gaussian distribution, periods with values exceeding 3σ were considered as times when anomalous values were observed, as they fell outside the range encompassing 99.7% of the data.

### Supplementary Information

Supplementary Information.
