# Supplementary material for: Detection of atmospheric radon concentration anomalies and their potential for earthquake prediction using Random Forest analysis
Source: Sci Rep. 2024 May 31;14:11626. doi: 10.1038/s41598-024-61887-6 (PMC11143197; doi:10.1038/s41598-024-61887-6)
Supplement: Supplementary file 1 — Supplementary Information. [file 41598_2024_61887_MOESM1_ESM.docx]

**Detection of atmospheric radon concentration anomalies and their potential for earthquake prediction using Random Forest analysis**

Mayu Tsuchiya¹，Hiroyuki Nagahama¹，Jun Muto¹，Mitsuhiro Hirano¹，Yumi Yasuoka²

1 Graduate School of Science, Tohoku University, Japan.

2 Radioisotope Research Center, Kobe Pharmaceutical University, Japan.


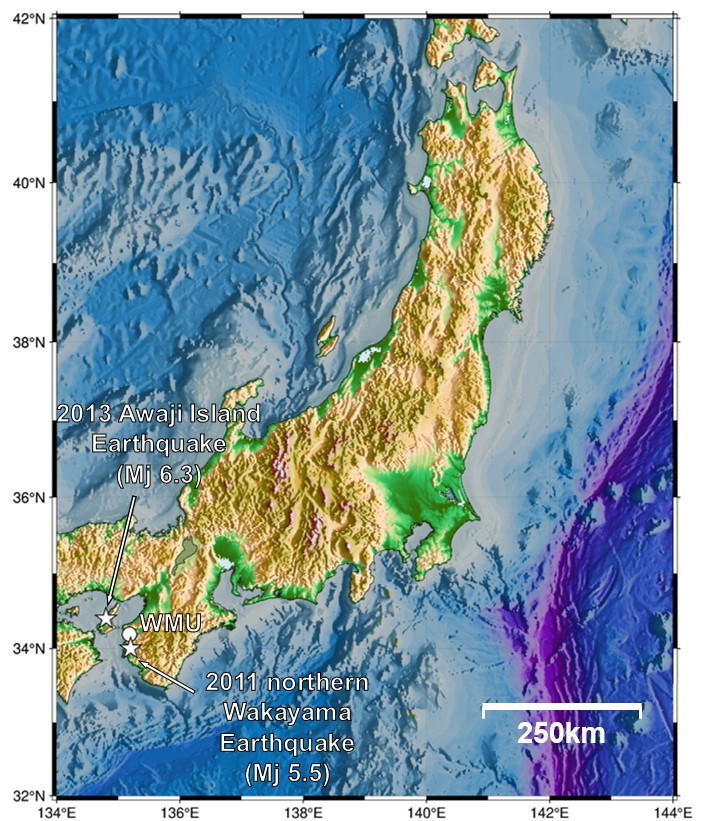


Supplementary Fig. S1. Map showing the locations where ionization current values were measured and the epicenters of the great earthquakes. The circles and stars indicate the location of Wakayama Medical University (WMU) at the observation site and the epicenters (the 2011 northern Wakayama Earthquake and the 2013 Awaji Island Earthquake), respectively. This map was created using PyGMT [40].


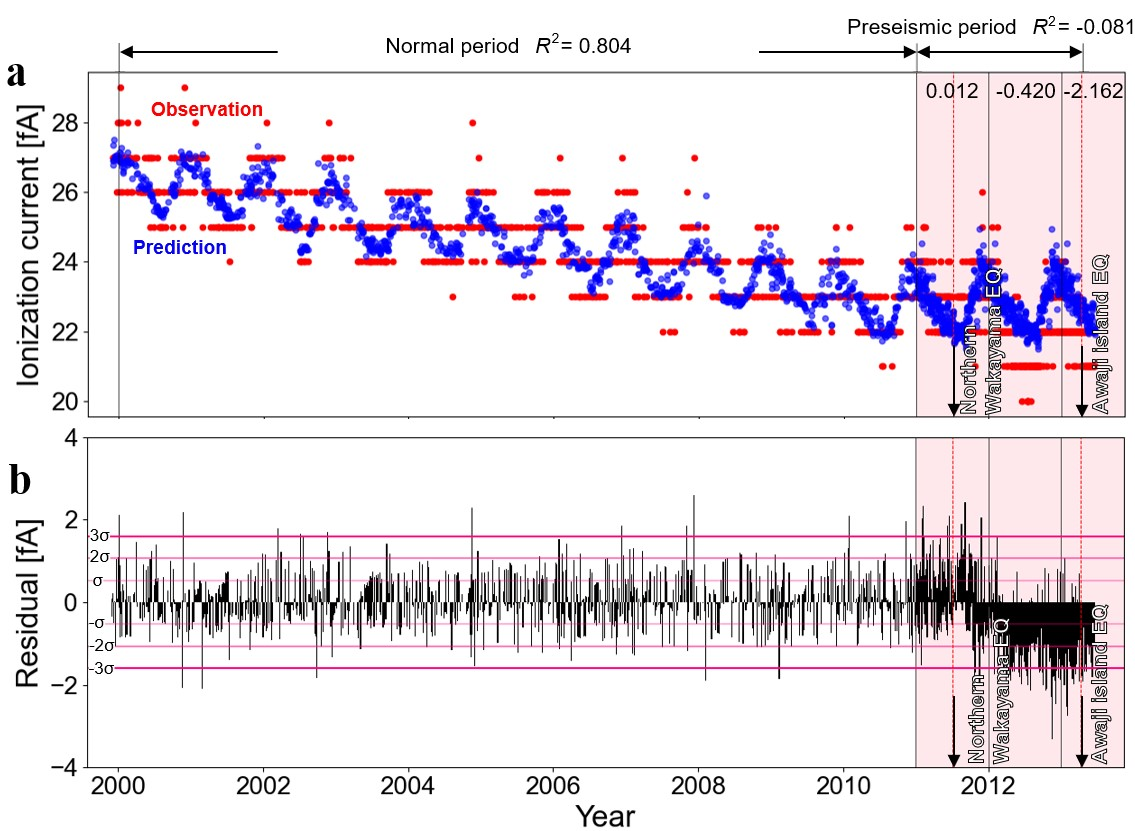


Supplementary Fig. S2. Prediction model and results in WMU. Red dots indicate actual ionization current values and blue dots indicate predictions obtained by Random Forest analysis. The coefficients of determination for each period are shown at the top. b) Discrepancy between observed and predicted ionization current values at WMU. Redish horizontal lines represent ±σ, 2σ and 3σ calculated from the standard deviation of the difference.

At WMU, in order to confirm that the timing of anomaly detection is in harmony with the previous study [9], the period used in the analysis was combined, with the normal period from December 1, 1999, to December 31, 2010, and the preseismic period from January 1, 2011, to June 20, 2013, as the data utilized in the analysis were the same as that used in the previous study. At WMU, data on fluctuations in atmospheric radon concentration during the earthquake swarm were observed, so they were included in the preseismic period. The data used in the analysis were observed in a gas flow-type ionization chamber installed in the RI facility at WMU [9]. We used a measuring instrument similar to FMU [7]. We conducted analysis using daily minimum values of ionization current measured hourly, similar to KPU and FMU.

Fig. S2 shows that compared to the KPU and FMU, periods of time when the difference between the observed and predicted values exceeds 3σ [17] occur more frequently before earthquake occurrence. This region is seismically active and has many cluster earthquakes. It is inferred that the frequency of exceeding 3σ is higher than that of KPU and FMU because there are more crustal deformations that cause fluctuations in atmospheric radon concentrations compared to KPU and FMU. In particular, prior to the July 5, 2011, earthquake in northern Wakayama Prefecture, the values exceeded 3σ continuously from June 9 to June 20, 2011. Also, before the April 13, 2013, Awaji Island earthquake, anomalies exceeding 3σ were continuously observed for a total of five times for 27 days from February 26 to the day of the earthquake.

Supplementary Table S1. Coefficient of determination for different depths of branches in Random Forests

|  | Unrestricted | 5 | 10 | 15 | 20 | 25 |
| --- | --- | --- | --- | --- | --- | --- |
| KPU | 0.813 | 0.743 | 0.816 | 0.813 | 0.813 | 0.813 |
| FMU | 0.718 | 0.706 | 0.727 | 0.719 | 0.718 | 0.718 |

The prediction model was created by running Random Forests with no depth restrictions and Random Forests of depths 5, 10, 15, 20, and 25. The depth with the highest coefficient of determination between the observed and predicted values was chosen. The table presents the coefficients of determination obtained at each depth. The top row of the table shows the depth of the branches in the random forest.

Supplementary Table S2. Coefficients of determination of the model calculated by cross-validation with 5 partitions of the normal period.

|  | 1 | 2 | 3 | 4 | 5 | Measurement error |
| --- | --- | --- | --- | --- | --- | --- |
| KPU | 0.834 | 0.848 | 0.848 | 0.850 | 0.821 | +/-0.005 |
| FMU | 0.732 | 0.649 | 0.713 | 0.664 | 0.739 | +/-0.018 |

We divided the normal period into five segments (1 ~ 5) and tested the coefficient of determination of the model by cross-validation. The top row shows the randomly divided segment numbers and the error ranges.
